# Rheological, baking, and sensory properties of composite bread dough with breadfruit (*Artocarpus communis* Forst) and wheat flours

**DOI:** 10.1002/fsn3.321

**Published:** 2015-12-16

**Authors:** Adegoke H. Bakare, Oluwatooyin F. Osundahunsi, Joseph O. Olusanya

**Affiliations:** ^1^ Department of Hospitality and TourismFederal University of AgricultureAbeokutaNigeria; ^2^ Department of Food Science and TechnologyFederal University of TechnologyAkureNigeria; ^3^ Department of Home Economics and Hotel ManagementTai Solarin University of EducationIjebu OdeNigeria

**Keywords:** Bread, production, rheology, sensory attributes qualities

## Abstract

The rheological (Pasting, farinograph, and alveograph) properties of wheat flour (WF) replaced with breadfruit four (05–40%) was analyzed. Baking and sensory qualities of the resulting bread were evaluated. Differences in baking properties of loaves produced under laboratory and industrial conditions were analyzed with *t*‐test, whereas ANOVA was used for other analyses. Peak and final viscosities in the composite blends (CB) ranged from 109.20 to 114.06 RVU and 111.86 to 134.40 RVU, respectively. Dough stability decreased from 9.15 to 0.78 min, whereas farinograph water absorption increased 59.7–65.9%. Alveograph curve configuration ratio increased from 1.27 to 7.39, whereas specific volume (Spv) of the loaves decreased from 2.96 to 1.32 cm^3^/g. The Spv of WF loaves were not significantly different (*P* > 0.05) from that of the 5% CB, whereas production conditions had no significant effects on absorbed water (*t* = 0.532, df = 18 *P* = 0.3005), weight loss during baking (*t* = 0.865, df = 18, *P* = 0.199), and Spv (*t* = 0.828, df = 14.17, *P* = 0.211). The sensory qualities of the 5% blend were not significantly different from the WF.

## Introduction

Bread is a staple food consumed in different parts of the World. Varieties of bread of different sizes, shape, textures, and flavors that contain different ingredients and are baked under different conditions exists across many continents (Zhou and Therdthai 2006). Bread was also described as a fermented confectionery product produced mainly from wheat flour, water, yeast, and salt by a series of process involving mixing, kneading, proofing, shaping, and baking (Dewettinck et al. 2008). Several studies have reported the prospects of composite flour technology as a means of reducing the dependence on wheat for the production of bakery products (Hugo et al. 2000; Doxastakis et al. 2002; Woo and Seib 2002; Hallen et al. 2004; Michiyo et al. 2004; Greene and Bovell‐Benjamin 2004; Mepba et al. 2007; Biljan and Bojana 2008; Manuel et al. 2008; Alex et al. 2008; Ade‐Omowaye et al. 2008; Lin et al. 2009; Adeleke and Odedeji 2010;. Malomo et al. 2011, 2013).

Consumption of wheat‐based products is expected to increase as population and urbanization increases (Bakare 2008; Malomo et al. 2011), and with the exotic lifestyle and food habits adopted by the citizen of most of the nonwheat producing countries. Partial as against the desired complete replacement of wheat flour with available indigenous flours remains the only viable practical option. The challenges with adoption of this technology at industrial scale include minimizing the changes that would occurred in the known quality of the final product, technical issues relating to the use of established manufacturing procedure and availability of flour of suitable quality from various indigenous sources; particularly flour from under utilised crops with less competing uses.

Breadfruit (*Artocarpus communis* Forst) is one of such crops. It is a staple diet in many tropical developing countries of the world. The tree fruits early between May and August, producing 50–200 fruits in a year. The mature fruit is round or ovoid, 15–20 cm in diameter and weighs 2–10 kg on average. The fruit is produced mainly in Malaysia, the South Pacific Island, the Caribbean's and West Africa (Ragone 2009). Total yearly production in Nigerian is about 10 million metric tonnes with potential to exceed 100 million metric tonnes with improved agricultural practice (Adewusi et al. 1995; Amusa et al. 2002). The economic utilization of breadfruit has been limited by its poor storage properties which is about 1–3 days after harvest (Ragone 2009). Conversion of breadfruit to flour would provide a more stable storage form and also enhance its versatility. (Morton 1987; Oladunjoye et al. 2010).

Breadfruit is an underutilized crop in Nigeria, information on its composition, nutritional importance, and the behavior of its flour during cooking had already been studied (Graham and De‐Bravo 1981; Oladunjoye et al. 2010; Bakare et al. 2012). This study therefore evaluated the effects of replacing wheat flour with breadfruit flour on the rheological properties of the composite flour blends, the baking and sensory qualities of the resulting bread and also compared some baking qualities of the bread under laboratory and industrial conditions.

## Materials and Methods

### Source of materials

Seedless variety of breadfruit (*Artocarpus communis* Forst) used for the study was obtained from a farm at Mamu in Ijebu North Local Government area of Ogun State. Wheat flour was obtained from Honey well flour mill, Ltd Apapa, Lagos, whereas other ingredients were purchased in the retail markets.

### Preparation of flour

Breadfruit was processed as described by Oladunjoye et al. (2010) with some modifications. Matured breadfruit (Greenish‐yellow skin), peel color index ‐ 3 (Ajayi 1997; Ragone 2009) were hand peeled, washed, thinly sliced, and soaked in 5% sodium metabisulfite solution for 30 min to prevent enzymic browning. It was then dried in a cross flow Gallenkamp oven (Model OV‐160 size 2 BS, Weiss Technik UK, Loughborough, Leicestershire, UK) at 80°C for 2 h and then, at 35°C for 12 h. The dried chips were milled to flour in a Disk mill (Model FFC‐15, Shandong‐Jimo Agricultural Machinery, Qingdao City, Shandong Province, China) at 8800 RPM and sieved through a 250 *μ*m mesh sieve (W. S. Tyler, 8570 Tyler Blvd, Mentor, OH, United States).

### Analysis of flour

All analyses were performed in triplicates. Protein, moisture, water absorption, and ash contents of wheat flour and blends were analyzed with official methods (AACC, 2000) using a Partens Inframatic analyzer (Model 9140, SE‐126 53, Hägersten, Sweden). Alpha‐amylase activity was determined with the Hagberg falling numbers instrument (Partens model no 1500, USA), based on (AACC, 2000) approved method. Free sugar and starch in wheat and breadfruit flours samples were determined by the spectrophotometric method of Dubois et al. (1970) and Mcready (1970). Crude fiber content of flour was determined by Trichloroacetic acid method as described by Entwistle and Hunter (1949).

### Viscosity characteristics of flour

Pasting characteristic of the four and their blends were determined according to ICC No 162 method. Rapid visco analyzer (RVA) series 4 (RVA; series 4, Newport Scientific P.T.V., Warriewood, Australia) with the aid of thermocline for Windows (version 1.1. Software, 1996) provided by the instrument manufacturer was used for the analysis. The 12‐min profile was used for all the analyses. It consists of idle temp of 50°C for 1 min, then 50–95°C in 3 min 45 sec, held at 95°C for 2 min 30 sec, cooled to 50°C over 3 min 45 sec, final, held at 50°C for 2 min. Two paddle speeds of 960 revolutions per minute for the first 10 sec followed by 150 revolutions per minute were employed for the remaining duration of the test cycle. The weight per sample used for each analysis was calculated from the formula:

$$\text{Corrected sample weight for R V A (S)}=\frac{A\;\times \;100}{100\;-\;M},$$

(W)=25+(A−S),

where *A *= Weight of flour sample: using the *R V A* manual as a guide.

*S *= Corrected sample weight.

*M *= Actual moisture content of the sample.

*W *= Volume of water used.

### Rheological characteristics of dough

#### Farinogram characteristics

The farinogram characteristics of dough made from blends of breadfruit/wheat composite Flour samples (10–20%) were determined by approved method (AACC, 2000) using Brabender Farinograph (Model T 150 E, Ohgduisburg, Germany). The instrument provided a means of evaluating the strength of flour, dough consistency, and characteristics (Edmund 1967; Bloksma 1990a; Abang Zaidel et al. 2010). Inframatic analyzer was used to obtain an estimate of the moisture content of flour sample and hence determine the actual weight of flour samples to be used at the prescribed 14% moisture basis for the farinogram analysis. This was obtained by the expression;

$$\text{Required weight of sample}=\frac{(100-14)\;\times \;300\;g}{\left(100.M\right)},$$

where *M *= Percent moisture content of the sample.

Appropriate weight of flour sample was placed in the mixer of the farinogram, which was thermostatically controlled by means of water jacket at a temperature of 30°C. Cold water at 30°C was added to the sample through the attached burette until optimum water absorption content was absorbed by the dough when the farinogram curve was on the 500 line. A fresh sample was taken and the process was repeated using the appropriate water absorption for the mixing and development process. The development of the dough and the resistance offered to mixing were recorded on the farinogram.

#### Alveogram characteristics

The alveograph (Chopin NG France) was used to measure (AACC, 2010) characteristics that provided insight in to the fermentation tolerance of the dough as may be exhibited during proofing stage of bread making. Characteristics of interest that were measured included the average resistance to expansion indicated by the peak height (mm), extensibility indicated by length (*L*) of the alveogram curve, energy input (Joules) required for the mechanical deformation of the dough (W), inflation required for maximum development (G), and the elastic resistance (Ie) of the measured dough samples.

Flour sample (250 g) of known moisture content was placed into the mixer, sodium chloride solution (2.5%) was added through a burette (i.e., 129.4 ml for flour with 14% moisture) and mixed for 7 min. The dough was forced through the extrusion gate in the form of a thin strip on to a small oiled steel plate. Five extruded dough pieces of designated length were cut off, rolled with an oiled rolling pin to a uniform thinness, cut into a circular disk, transferred to an oiled steel plate, and subjected to a brief rest period in a tempered compartment of the alveograph for 15 min. Each circular dough test pieces were removed from the compartment and inserted between two metal plates that held it securely in position. The air valve was opened to supply air pressure to the held dough through an orifice. The electrically driven recording manometer was simultaneously activated to record the air pressure inside the dough bubble against time.

#### Bread production

Bread loaves were produced according to AACC (2010) with slight modification. Formulation included: Breadfruit/Wheat (100:00, 90:10, 80:20, 70:30, and 60:40) composite Flour 100 g (14% moisture), 6.5 g sugar, 1.5 g iodized salt, 3.0 g yeast, 3.9 g fat and 50 ppm of ascorbic acid as dough improver. The water required to form a dough of desired consistency varied between 32.1% and 93.3% of flour using water absorption values obtained from the farinogram as guide.

Bread production was carried out in the laboratory and also in a small‐scale industrial bakery (Eucharistic Heart of Jesus, Ibonwon, Lagos state), respectively. Locally fabricated horizontal high speed mixer (Jido Nigeria) and Omega spiral mixer (Model OMJ‐25, China Omega Baking Machinery Co. L. No.88, East Taishan Road, Shenzhou City, Hengshui, Hebei, China) were used in the industrial and laboratory mixing, respectively. The dough was fermented (proofed) at initial and final fermentation time of 15 and 28 min and at ambient condition of 28 ± 2°C temperatures and 85 ± 12% relative humidity. Baking trials at laboratory level was done in an oven (Model GP‐OV‐100‐F‐SS‐DIG, St Helens, Merseyside, Great Britain) at 220°C for 30 min while locally constructed typical clay oven used by local bakeries was used for the industrial production. The bread samples were cooled for 1 h, then placed in low‐density polyethylene plastic bags and kept at 24 ± 2 °C.

#### Determination of physical quality of bread

Weights of bread loaves were measured with a Mettler Toledo (A204) digital weighing scale. Volume of was measured by millet seed displacement method (AACC, 2010) with minor modification.

#### Weight loss

The weight loss of the bread was determined as described by Kim et al. (2003). The dough was weighed before baking, and the breads were weighed after baking. The percent weight loss of the bread samples was calculated as:

$$\%\text{weight loss}=\frac{A-B\;\times \;100}{A},$$

where, *A* = weight of dough; *B* = weight of baked bread.

### Sensory analysis

#### Selection of panelists

Forty people were selected from a pool of volunteers comprising professional bakers, catering officers, lecturers, and students of tertiary institution. The panelists were selected after an oral interview conducted on the basis of a criteria checklist that included: Good health, nonsmoker, nonallergic to wheat/breadfruit, willingness to participate, and passion/likeness for the consumption of bread. They were compensated for their participation. Ten of the selected people were trained as panelists for descriptive analysis aspects of the test, whereas the remaining 30 people were used as untrained panelists for the consumer/preference aspect of the test.

#### Descriptive sensory analysis

Descriptive sensory analysis was carried out as described by Bakare et al. (2013). Judges rated the intensity of the samples for each attribute using a numerical intensity scale specified in Table 1

**Table 1:** Attributes, definitions, and references used in the descriptive sensory analysis of bread produced from wheat‐ breadfruit flour

Terms	Definitions	References	Quality and score range
Appearances			
Crust color	Light golden brown	100% wheat flour bread	Very pale dark brown to typical yellowish brown: 1–5
Crumb color	Typical white color of bread	100% wheat flour bread	Brown to white: 1–10
Cell size	Cell size of crumb resembling mesh of tiny diameter size	Internal crumb bread from 100% wheat flour.	More cells with small diameter: 1–10
Cell uniformity	Even distribution of the cells throughout the sample	Internal crumb bread from 100% wheat flour.	Extent to which cells are evenly distributed: 1–10
Flavor			
Characteristic taste of Bread	Reminiscent of the characteristic of typical wheat flour bread	Bread made with 100% wheat flour	Foreign to typical: 1–5
Wheaty smell	Reminiscent of the smell of wheat bread	Bread made with 100% wheat flour	Foreign to typical: 1–10
Aroma	Characteristic aroma of freshly baked bread	Bread made with 100% wheat flour	Foreign to typically pleasant: 1–5
Texture			
Mouthfeel	Having chewiness that is associated with freshly baked wheat flour bread	Bread made from 100% wheat flour	Doughy to typical clean easy to breakdown mouth feel: 1–10
Crumb stability and softness	Bread separated slowly when pulled apart	Bread made from 100% wheat flour	Unstable/hard to stable and soft: 1to 10
Nongrittiness	Absence of small coarse particles in the mouth after swallowing	Bread made from 100% wheat flour	Absence of small particles during and after mastication: 1–10
Lightness	Bread loaf has heavy, compact, thick inner structure associated with poorly aerated bread		Denseness to lightness: 1–10

Adapted from Greene and Bovell‐Benjamin (2004); Indrani and Rao (2007); Bakare (2008).

#### Consumer test

Quantitative acceptance test was used to assess consumers liking for the product. Thirty untrained panelists rated their liking or otherwise for cakes produced from the blends on a seven‐point hedonic scale (1 = liked very much less as compared to reference sample ‘*R*’, and 7 = liked much more as compared to reference sample ‘*R*’).

### Statistical analysis

All experiments and analyses were conducted in triplicates. Data obtained from different aspects of the study were subjected to analysis of variance and the Duncan multiple range test was used to separate the means (Duncan 1955). Independent sample *t*‐test was used compare the baking qualities (absorbed water, weight loss, and specific volume) of bread baked under laboratory and industrial conditions. Statistical analysis package software SPSS 17 for windows (IBM, New Orchard Road, Armonk, New York) was used for all the analyses.

## Results and Discussion

### Composition of the flours and their blends

The breadfruit flour (BF) had relatively lower protein but higher ash and fiber contents than wheat flour (Table 2) and did not have the structural gluten‐forming protein found in wheat flour. Protein and gluten contents of the blends decreased as wheat flour (WF) was gradually replaced with BF, whereas ash contents increased in the blends as the proportion of BF increased. Similar trend in protein valued was reported by Olatunji and Akinrele (1978). Values for protein and ash contents in the blends ranged from 6.71 to 11.1% and 0.83% to 1.41, respectively. Falling number values (which are indicative of the alpha amylase activity) increased from 316.3 to 865 as the proportion of BF was increased in the blends (Table 2). This implied that the extent of liquefaction and diastatic activity of the starches in the blends decreased as the proportion of the BF was increased (Schiller 1984; Watson 1984). The value of damaged starch in WF was within the range specified for bakery flour (Schiller 1984). The BF had a higher value of damaged starch (19.3%) than the WF. It was relatively higher than the value reported for breadfruit starch (Loos et al. 1981). The high fiber content (7.79%) observed in the BF suggested greater tendency to absorb more water during mixing than the WF and this has implication on the quality of bread produced from the blends.

**Table 2:** Composition of breadfruit, wheat flours, and their composite blends

Flour	Moisture (%)	Protein (%)	Ash (%)	Gluten (%)	Alpha amylase activity (Falling No)	Starch (%)	Damaged starch %	Sugars (%)	Fibers (%)
BF:WF									
00:100	12.5 ± 0.3^a^	10.9 ± 0.1_de_	0.65 ± .01_a_	12.8 ± 0.1_e_	370.3 ± 2_c_	69.89 ± 8	7.21 ± 0.1	2.63 ± 0.6	2.81 ± 0.7
100:00^#^	13.7 ± 0.2_b_	2.6 ± 0.1_a_	1.72 ± .01_f_	N.A	N.D	61.30 ± 4	19.3 ± 1.4	3.75 ± 0.7	7.79 ± 0.6
10:90	13.7 ± 0.2_b_	11.1 ± 0.3_e_	0.83 ± .0_b_	11.6 ± 0.1_d_	316.3 ± 0.7_b_				
20:80	12.8 ± 0.1_ba_	10.3 ± 0.3_d_	0.98 ± 0.0_c_	9.8 ± 0.1_c_	302 ± 1.4_a_				
30:60	12.5 ± 0.2_a_	8.59 ± 0.3_c_	1.31 ± .01_d_	7.6 ± 0.2_b_	686 ± 4.2_d_				
60:40	12.5 ± 0.1_a_	6.71 ± 0.1_b_	1.41 ± 01_e_	5.8 ± 0.1_a_	865 ± 2.1_e_				

a–f, Mean in the same column with the same subscripts are not significantly different (*P* < 0.05); N.D, Not determined; N.A, Not available; WF, wheat flour; BF, breadfruit flour; (10–40), composite blends; # = Substitution levels.

### Rheological characteristics

Rheology is the science of the deformation and flow of matter. It is the study of the manner in which materials respond to applied stress or strain (Mirsaeedghazi et al. 2008). The rheological properties of food materials measured or tested by rheometers like rapid visco anlyser, farinograph and alveograph provides empirical information that correlates well with actual results on product's quality. Also, measurements obtained from these instruments have been reported to correlates with result gotten from mixolab (Dapčević et al. 2009) which has been designed to eliminate some of the short comings associated with farinograph and amylograph (Alava et al. 2007; Fustier et al. 2008; Marco and Rosell 2008; Ozturk et al. 2008).

### Viscosity characteristic

Pasting temperature gives an indication of temperature required to cook the flour beyond its gelatinization point (BeMiller 2011). It corresponds to the temperature where viscosities first increase by at least 2 RVU over a 20 sec period (Olkku and Rha 1978; Appelqvist and Debet 1997). Changes that may occur when a starch‐water system is heated included enormous swelling, increased viscosity, translucency and solubility, and loss of anisotropy (birefringence) (Shimelis et al. 2006). The starch granules of the BF gelatinized at a higher temperature (71.7°C) than that of WF (68.50°C). The starch gelatinization range observed for wheat flour was within the range reported by Hoseney (1994) and Shimelis et al. (2006).

Peak viscosity is the maximum viscosity develops during or soon after the heating phase of the test. It occurred after most of the granule swelling had ceased. Hot starch paste is a mixture of swollen starch granules and granule fragments, together with colloidal and molecularly dispersed starch molecules. It gelatinizes when heated beyond 50°C. This caused a marked increase in the viscosity and further disintegration of the starch granules. The viscosity of the starch paste dropped at elevated temperature near 95 °C depicting the characteristic peak in the viscosity–temperature curve of the RVA graph (Dengate 1978; Bakare et al. 2012). The peak viscosity also measures the alpha amylase activity and other contributory factors such as the inherent susceptibility of the starch to amylase and the starch gel strength (Watson 1984; Meera 2010). Therefore, a higher value of RVU at the peak of the curve indicated a lower diastatic activity and vice versa (Schiller 1984). The peak viscosity of WF (101.0 RVU) was found to be significantly lower than that of BF (252.0 RVU) (Table 3) indicating a relatively higher diastatic activity and lower gel strength.

**Table 3:** Pasting characteristics of breadfruit and wheat flours

Flours	Peak viscosity (RVU)	Holding strength (RVU)	Breakdown viscosity (RVU)	Final viscosity (RVU)	Set back viscosity (RVU)	Peak time (min)	Pasting temperature °C
BF:WF							
00:100	101.17 ± 0.7_a_	64.33 ± 0.1_a_	36.83 ± 0.6_a_	114.92 ± 0.7_b_	50.58 ± 0.1_e_	6.20 ± 0.7_b_	68.50 ± 0.4_a_
100:00	251.90 ± 0.9_g_	191.60 ± 0.7_f_	60.94 ± 0.6_c_	316.00 ± 0.7_f_	126.0 ± 0.7_f_	4.60 ± 0.7_a_	71.74 ± 0.3_d_
05:95	109.20 ± 1.4_b_	66.14 ± 0.2_a_	43.06 ± 0.4_b_	111.86 ± 0.3_a_	45.70 ± 0.5_c_	6.22 ± 0.1_b_	68.77 ± 0.1_ab_
10:90	114.06 ± 0.7_c_	77.13 ± 0.9_b_	37.03 ± 0.5_a_	118.92 ± 0.7_c_	42.79 ± 0.6_ab_	6.19 ± 0.0_b_	69.50 ± 0.4_b_
15:85	115.8 ± 1.1_d_	79.43 ± 0.2_c_	36.46 ± 0.6_a_	123.44 ± 0.3_d_	43.01 ± 0.2_b_	6.19 ± 0.1_b_	70.12 ± 0.1_c_
20:80	119.02 ± 0.7_e_	82.15 ± 0.1_d_	36.87 ± 0.6_a_	124.09 ± 0.7_d_	41.94 ± 0.4_a_	6.16 ± 0.1_b_	70.46 ± 0.2_c_
40:60	122.30 ± 0.6_f_	85.40 ± 0.3_e_	36.93 ± 0.4_a_	134.40 ± 0.3_e_	48.37 ± 0.3_d_	6.07 ± 0.4_b_	78.30 ± 0.2_c_

a–g, Mean in same column with the same subscripts are not significantly different (*P* < 0.05); WF, wheat flour; BF, breadfruit flour; (05–90), composite blends.

Peak viscosities occur at equilibrium between swelling of the granules (that ‘increases the viscosity) and the granule rupture and alignment (that reduces viscosity). The relatively high swelling capacity exhibited by the BF may have resulted from a weak internal bonding in the starch granules.

Holding strength indicated the ability of the starch granules to maintain their gelatinized structure when the paste was held at 95°C for 2 min 30 sec under mechanical shearing stress. The BF had a holding strength value that was higher than that of the WF.

Breakdown viscosity is a measure of the degree of starch disintegration. It is an indication of hot paste stability of the starch. The smaller the breakdown viscosity, the higher the paste stability (Hugo et al. 2000; Bakare et al. 2012). The BF had significantly higher (60.9 RVU) breakdown viscosity value than the WF (36.8 RVU) indicating relatively lower hot paste stability.

Final viscosity is the section of the paste gel curve where the gelatinized dispersion of starch becomes viscoelastic on cooling resulting in the formation of a loose paste or gel. The BF had significantly higher final viscosity value (316 RVU) than the WF indicating that it formed a firmer gel after cooking and cooling.

Setback viscosity is the phase of the pasting curve after cooling the starches to 50°C. This stage involved re‐association, retrogradation, or reordering of starch molecules. Also, the water previously bounded in the viscoelastic gel are released at this stage in a process referred to as syneresis. The higher the setback viscosity, the greater the tendency toward retrogradation. The BF had relatively higher viscosity value than the WF.

Peak time was the time at which the peak viscosity was attained in minutes. The WF had a significantly higher peak time than the BF.

### Farinograph

Mixing, fermentation and baking are the three basic operations involve in bread making. Mixing transforms the flour and water into cohesive viscoelastic dough and also incorporates air in to the dough. The incorporated air provided the gas cells into which the carbon dioxide produced by the yeast fermentation diffuses. Bread dough is a wet mass developed after mixing of wheat flour, water and other ingredients. Development of dough occurs as a result of interactions among flour constituents during mixing operation. Although, these interactions are more complex than what was observed during the farigraph test. The test, however, provided an empirically verifiable insight in to what may be at play during the actual process of dough development. The aim of mixing is to bring about changes in the physical properties of the dough that would improve the ability of the dough to retain the carbon dioxide gas that would be produced during yeast fermentation. Resistance to deformation, extensibility, elasticity, and stickiness are some of the physical properties of dough that are critical for control in bread making process.

The rheological characteristics exhibited by flour during mixing (Table 4) revealed that the WF arrived at the consistency line in 1.85 min, whereas the blends arrived at relatively short times, indicating faster uptake of water and faster dough development (Lorenz 1990). Arrival time, (AT) was the time to the nearest one‐half minutes required for the top of the curve to reach the point of greatest torque after the commencement of mixing (500 BU consistency line). It is a measure of the rate at which water was taken up by the flour (Shuey 1990; Abang Zaidel et al. 2010). Departure time (DT) was the time required for the curve to drop below the 500 BU consistency line. All the blends of BF and WF had shorter DT times compared to the WF.

**Table 4:** Farinogram of wheat flour and its composite blends with breadfruit flour

Flours	Arrival time (min)	Departure time (min)	Dough stability (min)	Mixing tolerance index (BU)	Water absorption (%)	Breakdown time (min)	Dough development time (min)
BF:WF							
00:100	1.86 ± 0.1_e_	10.9 ± 0.1_d_	9.35 ± 0.4_e_	80.5 ± 0.3_a_	58.6 ± 0.4_a_	10.9 ± 0.5_c_	8.25 ± 0.1_c_
05:90	1.69 ± 0.1_d_	10.6 ± 0.1_d_	9.15 ± 0.2_d_	82.5 ± 0.7_a_	59.7 ± 0.4_a_	10.7 ± 0.4_c_	8.28 ± 0.1_c_
10:90	1.04 ± 0.1_a_	3.44 ± 0.1_c_	2.25 ± 0.2_b_	106.5 ± 0.9_b_	64.7 ± 0.6_b_	2.56 ± 0.2_a_	1.65 ± 0.3_a_
15:85	1.06 ± 0.7_b_	3.13 ± 0.4_b_	2.36 ± 0.1_c_	106.80 ± 1.3_b_	65.5 ± 0.4_c_	2.48 ± 0.1_a_	1.63 ± 0.2_a_
20:80	1.67 ± 0.1_c_	2.54 ± 0.4_a_	0.78 ± 0.1_a_	161.7 ± 1.2_c_	65.9 ± 0.6_c_	2.65 ± 0.1_b_	2.15 ± 0.1_b_

d–a, Mean in same column with the same letter are not significantly different (*P* < 0.05); WF, wheat flour; BF, breadfruit flour; (05–20), composite blends.

Dough Stability Time (DST), indicated how much tolerance the flour has to over or under mixing (Schiller 1984). The WF had a DST value of 9.30 min, whereas all the blends have significantly lower DST which decreased as WF was replaced with BF. The DST values of the blends ranged from 0.78 to 9.15 min. This DST trends agreed with the reports of Olatunji and Akinrele (1978) for tropical tuber and breadfruit and Michiyo et al. (2004) for pre‐germinated and brown rice.

Water is responsible in hydrating the protein fibrils and facilitating the interactions between the proteins cross‐links with the disulfide bonds during dough mixing. An optimum amount of water is needed to develop cohesive, viscoelastic dough with optimum gluten strength. Optimum water level differs from flour to flour depending on the quantity of protein and other dense particles that they contained. Protein content has been known as an important determinant of the extent to which WF would absorbed water during mixing (Sliwinski et al. 2004). In composite flour, the influence of starches, fiber from nonwheat source, and relatively higher damaged starch in the BF on the absorbed water may be more significant than the effect of protein content in the flour blends as substitution of WF for BF progresses.

The water absorption is the amount of water required to develop dough to the point of greatest torque when, for wheat flour, the gluten would have been fully developed. The water absorption values ranged from 58.6 to 65.9% with the WF and the 20% blend having the lowest and highest values, respectively. Earlier studies (Doxastakis et al. 2002; Malomo et al. 2011) have also reported the absorption of more water by composite blends. The increases in water absorption values as the BF replaces WF in the blends may not be unconnected with the higher crude fiber content in the BF (7.8%) compared to the WF (2.81%), respectively (Table 3). Crude fiber have components that are hydrophilic (D'Appolonia and Kim 1976; Hu et al. 2007) and capable forming solution of high viscosity (Yin et al. 2011).

Mixing tolerance index (MTI) values ranged from 80.5 to 161.7 BU with the WF having the lowest values. It also decreased as BF was used to replace WF in the blends. Generally, flours with good tolerance to mixing have low MTI; the higher the MTI value, the weaker the flour (Shuey 1990). Breakdown Time (TBD) like MTI it is also an index of the relative strength of flours. The TBD values ranged from 2.65 to 10.9 min. The WF showed better resilience than the blends of composite flour.

**Figure 1:**
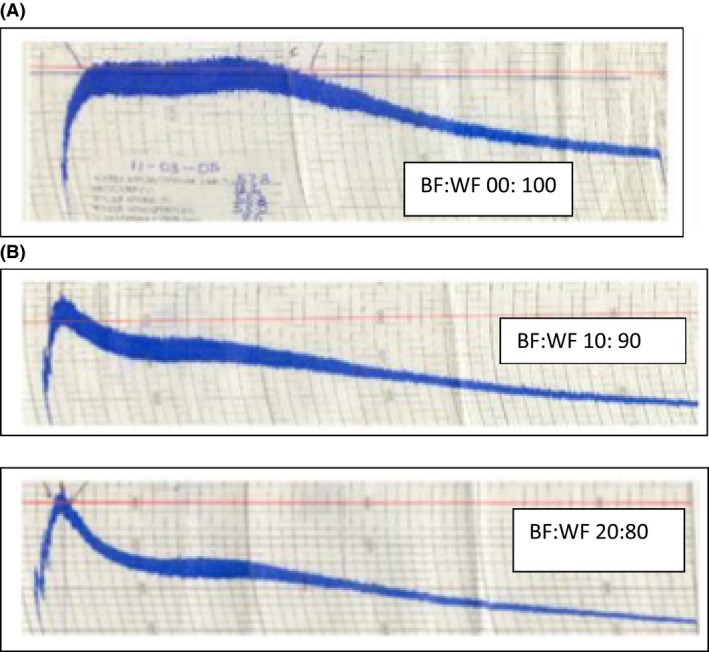
(A) Farinogram of Wheat and Breadfruit‐Wheat composite flour. (B) Farinogram of Wheat and Breadfruit‐Wheat composite flour.

Dough development particularly for the WF begins with addition of water and commencement of mixing operation. Initially all ingredients are hydrated and appeared like a sticky paste. Belton (1999) and Letang et al. (1999) demonstrated that gluten development was mainly brought about by the interactions of glutenin proteins with each other in the loop by disulfide bonds. On further mixing, more protein becomes hydrated and the glutenins tend to align because of the imposed shear and stretching forces (Abang Zaidel et al. 2010). the viscosity also increased, sticky characteristics of dough disappeared and a nonsticky mass was developed at peak consistency of dough typified as the peak of the curve above the 500 BU of the farinogram (Fig. 1A). The interactions between the polymers cross‐links was stronger and led to an increase in dough strength, maximum resistance to extension and restoring force after deformation. When the dough was mixed beyond its peak development, the cross‐links began to break due to the breaking of disulfide bonds. The glutenins become depolymerized and the dough is overmixed. The monomeric proteins, gliadins form a matrix within the long polymer networks and contribute to resistance to extension by forming viscous dough with reduced elasticity. The presence of smaller chains in the dough makes the dough stickier. In Figure 1B, the ability of the blends to sustain the viscoelastic property of the dough reduced with increasing presence of the BF as shown in the trends in dough stability, breakdown, and dough development time.

### Alveograph

**Figure 2:**
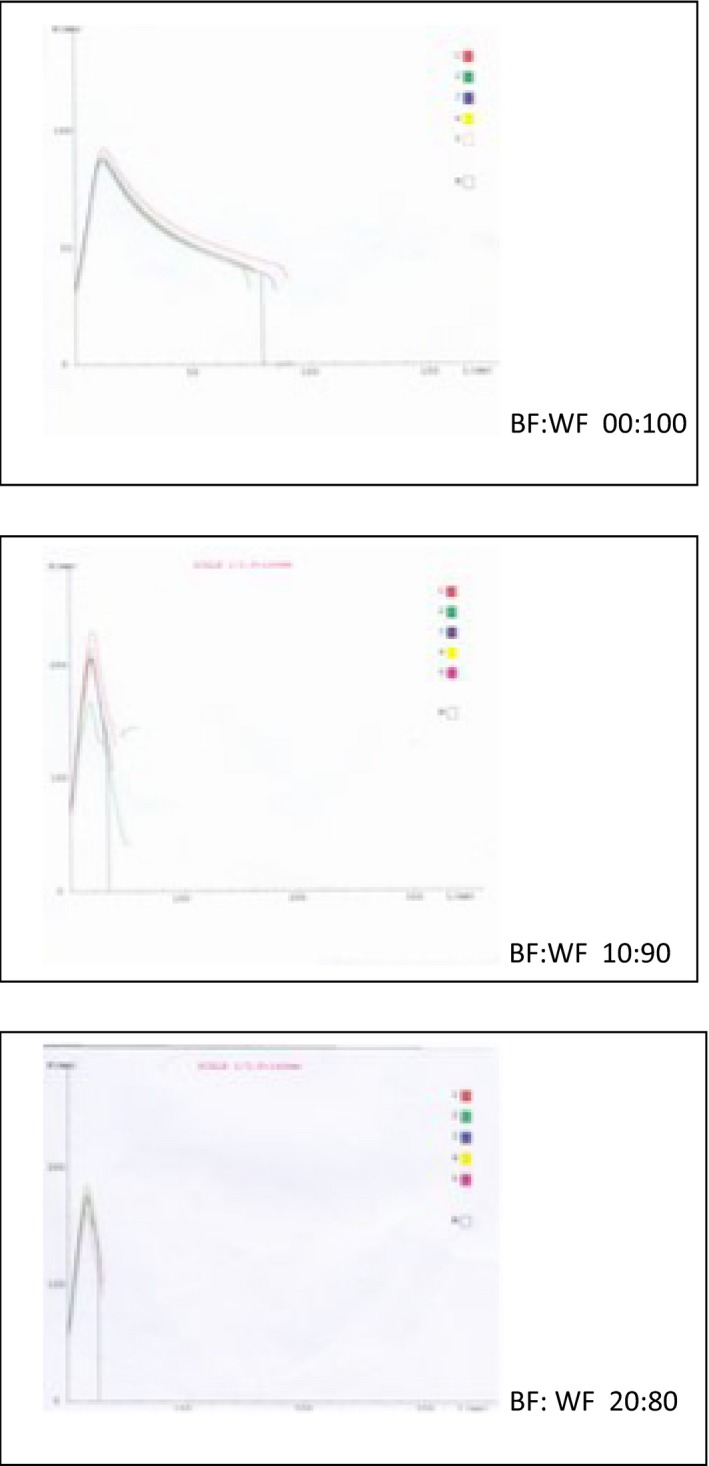
Alveogram of Wheat and Breadfruit‐Wheat composite flour.

The alveograph is an important dough testing instrument use to evaluate the quality of wheat flours for bread and biscuit and cookie making (Bettge et al. 1989; Janssen et al. 1996). It measures the resistance to expansion and the extensibility of a dough by providing the measurement for maximum over pressure, average abscissa at rupture, index of swelling, and deformation energy (Fig. 2) of dough (Indrani and Rao 2007). It impacts strain rates of 0.1–1 sec^−1^, which are about 100‐fold higher than those occurring in actual baking processes (Chin and Campbell 2005).

The Peak height (P) indicated the resistance that the dough offered to deformation and it is related the tensile strength or stability that the dough exhibited during the proofing stage of bread making (Pyler 1988; Mepba et al. 2007). The *P* values ranged from 90 to 226 mm with the WF and the 10% blend offering the least and highest resistance to expansion, respectively (Table 5). The length (*L*) indicated the extensibility of the dough. The *L* values ranged from 33 to 80 mm with the 10% blend and WF having the least and highest extensibility, respectively. The P/*L* (configuration ratio) ranged from 1.13 to 7.39. The WF and 20% had the highest and lowest values, respectively, and the 5% blend was not significantly (*P* < 0. 05) different from the WF.

**Table 5:** Alveogram of wheat flour and its composite blends with breadfruit flour

Flours	Peak height (mm) (P)	Length (mm) (L)	Energy (10^−4^J) (W)	Curve configuration (P/L)	Maximum inflation (G)	Elasticity index (%) (Ie)
BF:WF						
00:100	90 ± 0.4^a^	80 ± 1.4^d^	304 ± 0.7^c^	1.13 ± 0.2^a^	19.9 ± 0.6^b^	64.6 ± 0.4^e^
05:95	96 ± 0.7^a^	76 ± 0.7^c^	304 ± 1.4^c^	1.27 ± 0.4^a^	18.4 ± 0.4^b^	62.6 ± 0.4^d^
10:90	226 ± 0.7^d^	33 ± 0.7^b^	336 ± 2.8^d^	6.79 ± 0.3^b^	12.8 ± 0.4^a^	59.1 ± 1.4^c^
15:85	217 ± 0.6^c^	27 ± 0^a^	298 ± 1.4^b^	8.04 ± 0.2^d^	12.1 ± 0.2^a^	45.0 ± 0.7^b^
20:80	193 ± 0.7^b^	26 ± 0.7^a^	227 ± 1.4^a^	7.39 ± 0.2^c^	11.4 ± 0.4^a^	0.0^a^

d–a, Mean in same column with the same superscripts are not significantly different (*P* < 0.05); WF, wheat flour; BF, breadfruit flour; (05–20), composite blends.

The energy (*W*) required for deformation is an indication of the baking strength of the dough. It ranged from 227 × 10^−4^ J in the 20% blend to 336 × 10^−4^ J in the 10% blend. Baking strength increased as WF was replaced with BF and peaked at 10% substitution level which was the inflection point in the trend.

The curve configuration ratio (P/*L*) is an index of gluten behavior. It ranged from 1.13 to 8.04 with the WF and the 15% blend having the lowest and highest values, respectively. Also, there was no significant (*P* < 0.05) difference between the WF and 5% blend. High values of curve configuration ratio may be indicated strong wheat flour as observed by Pyler (1988). However, the strength of composite flour is probably influenced by considerations other than gluten behavior. The significantly higher ratios of the 10–20% blend could not be due their gluten contents alone. This G values (Table 5) depicted the relative abilities of the dough to be inflated for maximum development until they eventually burst. It is a measure of the magnitude of the total response of the dough to the biaxial stress and strain imposed on it by the instrument. It ranged from 11.4 to 19.9, decreased significantly as WF was replaced with BF. The WF and 5% blend had significantly higher values than other blends. Elasticity index (Ie) may be used to characterize the dough on the basis of the elastic resistance that they offer during their bi‐axial deformation (Pyler 1988; BaNu et al. 2011). It also decreased with substitution levels and the WF and 5% blend offered significantly better elastic resistance.

The nonlinear viscoelastic behavior of WF dough has been attributed to the continuous gluten matrix and starch granules embedded in it (Collar et al. 2007). It possesses the properties of both solid and liquid bodies, and exhibited the rheological properties that were in between that of the ideal solid and fluid bodies. The inclusion of BF in the blends increased the starch and fiber contents, whereas decreasing the quantity and quality of protein needed to sustain the viscoelastic behavior of their dough. These effects were aptly described by alveograph (Fig. 2) as shown by the significant (*P* < 0.05) decline of important quality indices (Table 5).

### Evaluation of bread quality

#### Absorbed water by dough

The quantity of water used to form dough of consistent quality (Table 6) for baking was significantly (*t* = 0.540, df = 13.89, *P* = 0.299) lower than the farinograph water absorption (Table 4). Similar observation was made by El‐Dash et al.*,* (1977) and Shuey (1990). This indicated that the presence of other baking ingredients may have been responsible for the reduction in the actual quantity of water used to form the dough.

**Table 6:** Physical quality characteristics of bread

Flour	Absorbed water (%)	Weight loss (%)	Specific volume (cm^3^/g)
BF:WF			
00:100	32.1 ± 0.4^a^	2.44 ± 0.3^a^	3.00 ± 0.05^d^
05:95	37.3 ± 0.3^a^	1.58 ± 0.1^a^	2.96 ± 0.01^d^
10:90	54.3 ± 0.9^b^	14.9 ± 0.6^b^	2.16 ± 0.04^b^
15:85	59.1 ± 0.2^c^	14.6 ± 0.6^b^	2.17 ± 0.04^b^
20:80	67.3 ± 0.1^d^	16.4 ± 0.4^c^	1.75 ± 0.05^a^
30:70	75.3 ± 0.5^e^	18.1 ± 0.5^d^	1.44 ± 0.03^a^
40:60	93.3 ± 0.9^f^	15.4 ± 0.5^b^	1.32 ± 0.02^a^

f–a, Mean in the same column with the same superscripts are not significantly different (*P* < 0.05); WF, wheat flour; BF, breadfruit flour; (05–40), BF/WF composite blends.

Absorbed water ranged from 32.1 to 99.3% with the WF and 40% blend having the highest and lowest values, respectively. The WF was not significantly (*P* > 0.05) different from the 5% blend but the both of them were significantly (*P* < 0.05) different from the rest of the blends. The significantly higher values of absorbed water in the blends when substitution was beyond 10% levels may be due to the relatively higher starch and fiber contents that may be present in the blends as the WF was gradually replaced by the BF.

#### Specific volume

Loaf volume is used as a criterion to measure the quality of fresh bread in research quality control in industry and by consumers (Penfield and Campbell 1990; Zuwariah and Noor Aziah 2009). Specific volume of loaves of bread provide a uniform basis for comparing results of various studies (Oyeku et al. 2008). It ranged from 1.32 to 3.00 cm^3^/g. The WF and 40% blend have the highest and lowest values. The values decreased as BF replaces the WF in the blends but the 5% blend was not significantly (*P* > 0.05) different from the WF.

Specific volume is an indication of the gluten content of the bread (Van Hall 2000; Abang Zaidel et al. 2010) but other constituents such as starch and fiber also contribute to the specific volume of bread. Gluten or more precisely glutenin, is the main structure‐forming protein in wheat flour that is responsible for the elastic and extensible properties needed to produce good quality wheat bread (Bloksma 1990b; : Gallagher et al. 2003). Bread made from soft wheat flour usually yield lower loaf volumes. It has also been shown that the difference between weak and strong flours can be explained by differences in the molecular mass distribution of their proteins (MacRitchie 1973). Abundance of glutenin molecules with long chain was observed to have made the protein phase, and consequently the dough, highly extensible (Bloksma 1990a).

Differences in the pasting temperature and peak viscosity of composite starches have been suspected to influence extensibility (Greene and Bovell‐Benjamin 2004). Pasting temperature is related to gelatinization temperature because it occurred after gelatinization. It was noted (Table 3) that pasting temperature increased as the WF was replaced with the BF. This implied that peak viscosity of each of the composite blend were attained at different pasting temperatures and may have induced additional tensile stress in dough membranes during baking. This may have over stretched the membranes beyond its capacity, ruptured it and terminating oven rise prematurely.

The differences in specific volume of the composite blend and the WF could therefore be traced to factors (Composition of the flour, their rheological and pasting properties) that directly determines their behavior during processing rather than the behavior of the dough themselves (Bloksma 1990b), because these are factors that influenced the specific volume.

#### Weight loss

Cut‐out dough losses weight during the proofing and baking stages of bread processing. This may be may be due to both fermentation losses brought about by amylases of starch and utilization of soluble sugar by yeast and also by evaporation of moisture during baking. Weight loss decreased as the BF replaces the WF in the blends (Table 6). It ranged from 1.68 to 18.1% with the 5 and 30% blend having the lowest and highest losses, respectively. The weight loss recorded for the 5% blend was not significantly different from the WF. The 20% was significantly different from the 30% blend and both have significantly higher weight loss values than other blends.

Significantly higher weight loss by the blends (except at 5%) could be attributed to their ability to form a viscous dough while imbibing large amount of water (Tables 4 and 6) which were lost during the baking.

#### Comparison of baking qualities of bread from laboratory and industrial conditions

Relatively more water was absorbed by the dough, greater weight losses were observed and higher specific volumes were recorded in the bread produced under industrial condition (Table 7). This may be due to the high amount of mechanical energy inputted by the locally fabricated horizontal high‐speed mixer and humidity condition of the oven. Results of independent *t*‐test for absorbed water (*t* = 0.532, df = 18, *P* = 0.3005, one‐tailed), weight loss (*t* = 0.865, df = 18, *P* = 0.199, one tailed), and specific volume (*t* = 0.828, df = 14.17, *P* = 0.211, one‐tailed), however, indicated there was no significant (*P* > 0.05) difference (0.601, 0.398, and 0.421 were >0.05) in the mean values of these quality parameters, respectively. These implied that there may be no technical hindrance to successful industrialization of this technology (Table 7).

**Table 7:** Comparison of baking qualities of bread produced under laboratory and industrial conditions

Blends	Absorbed water (%) L	Absorbed water (%) I	Weight loss (%) L	Weight loss (%) I	Specific volume (cm^3^/g) L	Specific volume (cm^3^/g) I
BF:WF 0:100	32.1	26.1	2.48	3.23	3.00	3.70
BF:WF 05:95	37.3	38.1	1.58	4.71	2.96	3.51
BF:WF 10:90	54.3	42.3	14.9	15.7	2.16	3.31
BF:WF 15:85	59.1	47.4	14.6	15.2	2.17	2.58
BF:WF 20:80	67.3	56.3	16.4	17.2	1.75	2.15
BF:WF 30:70	75.3	81.6	18.1	21.1	1.44	1.12
BF:WF 40:60^#^	93.3	88.3	15.4	21.6	1.32	1.1

L, laboratory; I, industrial**;** WF, wheat flour; BF, breadfruit flour; (05–40), BF/WF composite blends; # = Substitution levels.

### Descriptive sensory quality

#### Sensory quality of breads

**Figure 3:**
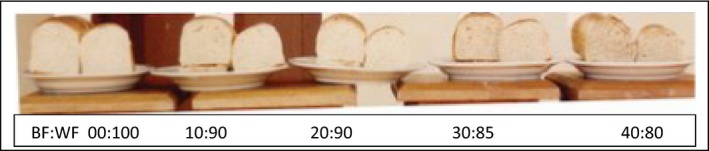
Samples of Wheat and Breadfruit‐Wheat composite flour.

The summary of the descriptive sensory attributes of the bread samples is presented in Table 8. The mean scores (3.55–6.73) for appearance (crust and crumb color, contour consistency, and grain quality) of the bread samples decreased significantly (*P* < 0.05) as the WF was replaced by the BF. The 5% blend was appreciated better than other blends in terms of appearance. Similar trends were observed for flavor (3.47–7.80) and texture (4.25–8.03). However, the 5% blend was not significantly different from the WF (Fig. 3).

**Table 8:** Sensory qualities of bread

Blends	SCORES	BF:WF/00:100	05:90	10:90	15:85	20:80	30:70	40:60
Attributes								
Appearance								
Crust color	5	3.0	3.5	3.3	3.3	3.3	2.5	1.5
Crumb color	10	7.0	7.3	5.5	6.0	5.0	5.5	3.7
Cell size	10	8.0	8.0	5.0	5.6	4.8	4.3	4.7
Cell uniformity	10	8.0	8.1	5.2	5.1	4.8	5.0	4.3
Mean		6.5 ± 2.4^d^	6.73 ± 2.2^d^	4.75 ± 1.0^c^	5.0 ± 1.2^c^	4.48 ± 0.8^ab^	4.33 ± 1.3^ab^	3.55 ± 1.4^a^
Flavor								
Taste	5	5.0	4.7	3.5	3.5	2.8	2.8	2.7
Wheaty smell	10	10.0	9.6	5.0	5.1	4.7	4.3	4.2
Aroma	10	8.0	8.5	4.5	4.7	3.5	4.5	3.5
Mean		7.67 ± 2.5^b^	7.80 ± 2.6^b^	4.33 ± 0.8^a^	4.4 ± 0.8^a^	3.67 ± 1.0^a^	3.87 ± 0.9^a^	3.47 ± 0.8^a^
Texture								
Mouthfeel	10	8.0	8.4	6.5	6.5	5.8	4.7	4.5
Crumb stability and softness	10	8.0	8.0	6.0	5.2	5.2	5.2	4.5
Grittiness	10	7.0	7.2	6.0	5.0	5.2	4.3	3.5
Lightness	10	8.0	8.5	6.0	5.5	5.2	5.2	4.5
Mean		7.75 ± 0.5^d^	8.03 ± 0.6^d^	6.12 ± 0.3^c^	5.55 ± 0.7^bc^	5.35 ± 0.3^b^	4.85 ± 0.4^ab^	4.25 ± 1.9^a^
General acceptance								
		7.85^c^	8.1^c^	7.04^c^	7.01^c^	5.83^b^	5.20^b^	3.71^a^

d–a, Mean in same column with the same alphabets are not significantly different (*P* < 0.05); WF, wheat flour; BF, breadfruit flour; (05–40), BF/WF composite blends.

#### Consumer acceptance

The mean score for consumer acceptance of the bread samples ranged from 3.71 to 8.10 with the 5 and 40% blends having the highest and lowest values, respectively. The panelists felt that bread samples produced when the WF was replaced with the BF up to 15%, was not significantly (*P* < 0.05) different from samples from the WF in terms of acceptance ratings.

## Conclusion

In this study, attempt was made to evaluate the extent to which WF can be replaced with BF in the production of bread and to assess whether such efforts could be replicated at industrial scale. This was done by analyzing the rheological properties of the flour and dough, as well as baking qualities of the resulting bread. Nigeria bakers have often complained about the gaps between research results obtained in the laboratory and the practicality of such results at industrial level. Attempt was made in this study to bridge such gap by comparing laboratory baking test result with that at industrial level.

The study concluded that the foam structure of bread crumbs was determined by the network of gelatinized dispersion of starch interwoven with strands of gluten. Gluten, the main structure‐forming protein in WF that was responsible for the elastic and extensible properties needed to produce good quality bread decreased as the WF was replaced with the BF. Interactions between gluten (specifically gliadin), starch, and other components of the flour were responsible for the viscosity properties that contributed to the aeration of the dough. Ability of the dough to sustain this aeration particularly during oven rise under baking condition was determined in the blends, by their relative viscosity properties.

The BF that was used to replace the WF had lower starch but higher fiber contents. These therefore altered the pasting characteristics and other rheological (farinograph and alveograph) properties of the blends especially at substitution levels beyond 5% and made them to be significantly different from that of WF. These also resulted in adverse changes in baking qualities. Bread sample whose specific volume was not statistically different from that of WF was only obtained at 5% level of replacement of the WF with BF. The objectivity of these was further reinforced as panelists were unable to significantly detect differences in sensory attributes between the samples from the 5% blend and those from WF. Although, panelist felt that bread of acceptable sensory quality similar to that produced from the WF was obtained within 15% substitution levels. In order to prevent serious deviation from the widely accepted quality attributes of bread and also for ease of adapting the technology to industrial level, it is recommended that substitution should be limited to a range from 5 to 10%. More so, when results have showed that this effort can be replicated at industrial level without significant alteration in baking quality indices.

## Conflict of Interest

None declared.
